# Supplementary material for: Intricacies of human–AI interaction in dynamic decision-making for precision oncology
Source: Nat Commun. 2025 Jan 29;16:1138. doi: 10.1038/s41467-024-55259-x (PMC11779952; doi:10.1038/s41467-024-55259-x)
Supplement: Supplementary file 1 — Supplementary Information [file 41467_2024_55259_MOESM1_ESM.pdf]

Supplementary Information

Intricacies of Human-AI Interaction in Dynamic Decision-Making for Precision Oncology

Niraula et al.

Contents

Supplementary Table 1: Patient's Clinical Information 1
Supplementary Figure 1: Snippets of Unassisted page treatment plan viewer. 2
Supplementary Figure 2. Snippets of Unassisted page PET/MRI viewer 3
Supplementary Figure 3. Snippets of AI-assisted Pages showing ARCliDS recommendation, estimated outcome, and feature distribution panels 4
Supplementary Table 2: List of Software and Library 5
Supplementary Table 3: Evaluators Summary 6
Supplementary Figure 4: Decision Adjustment Level 7
Supplementary Figure 5: Correlation between Unassisted and AI-assisted Decision and Confidence. 8
Supplementary Figure 6: Individual AI-Trust Level Contribution vs Agreement with AI-recommendation 9
Supplementary Figure 7: Authors summary of Evaluators' Remark for NSCLC. 10
Supplementary Figure 8: Authors summary of Evaluators' Remark for HCC. 11

Supplementary Table 1: Patient's Clinical Information

NSCLC											
**Pat No**	**Sex**	**Age**	**Cancer Stage**	**Smoking History**	**COPD**	**CVD**	**Hypertension**	**Histology**	**Chemo**	**KPS**	**GTV [cc]**
1	Female	56-60	1	Yes	No	No	No	Poorly Differentiated	Yes	90	19
2	Male	56-60	3	Yes	No	No	Yes	Adenocarcinoma	Yes	100	207
3	Female	81-85	3	No	No	No	No	Adenocarcinoma	Yes	80	19
4	Female	56-60	3	Yes	No	No	Yes	Adenocarcinoma	Yes	90	58
5	Male	56-60	3	Yes	No	No	Yes	Squamous Cell Carcinoma	Yes	80	359
6	Female	56-60	3	Yes	No	No	No	Adenocarcinoma	Yes	90	102
7	Male	56-60	3	Yes	No	No	No	Squamous Cell Carcinoma	Yes	90	180
8	Female	61-65	3	Yes	Yes	No	No	Squamous Cell Carcinoma	Yes	70	113

HCC													
**Pat No**	**Sex**	**Age**	**Cirrhosis**	**No of Active lesion**	**PVT**	**No of Pre-SBRT Systemic Therapies**	**No of Pre-SBRT Liver-Directed Therapies**	**Presence of Extrahepatic Disease**	**No of Prior Liver Occurrences**	**Previously Treated**	**ECOG-PS**	**GTV [cc]**	**Liver minus GTV [cc]**
1	Female	76-80	Yes	1	No	0	2	No	1	Yes	0	17.02	1641
2	Female	76-80	Yes	2	No	0	4	No	3	Yes	0	0.75, 0.92	2546
3	Female	71-75	Yes	4	No	0	2	No	2	Yes	1	4.32, 1.92, 6.32, 0.56	1346
4	Female	76-80	Yes	1	No	0	2	No	2	No	1	2.2	1416
5	Male	56-60	Yes	1	No	0	2	No	3	No	0	9.9	1714
6	Female	56-60	Yes	1	No	0	3	No	1	Yes	1	4.3	2287
7	Female	56-60	Yes	1	Yes	0	0	No	0	No	0	469	1604
8	Female	76-80	Yes	1	No	0	5	No	4	Yes	0	5.51	1281
9	Female	61-65	Yes	1	No	0	1	No	0	No	0	2.04	1533

Supplementary Table 1: Patient's clinical information provided to the Evaluators. For NSCLC, the columns names are patient's sex, age (exact age was provided during evaluation), cancer stage, smoking history (binary), chronic obstructive pulmonary disease status (COPD, binary), cardiovascular disease status (CVD, binary), hypertension status (binary), histology (categorical), chemo status (binary), Karnofsky performance status (KPS), and gross tumor volume (GTV, in cc). For HCC, the column names are patient's sex, age, cirrhosis status (binary), number of active lesions, portal vein thrombosis (PVT) status (binary), number of pre-SBRT line of systemic therapies, number of pre-SBRT liver-directed therapy, presence of extrahepatic disease status (binary), number of prior liver occurrence, previous treatment status (binary), eastern cooperative oncology performance status (ECOG-PS), gross tumor volume (GTV, in cc), and Liver minus GTV (in cc).

Supplementary Figure 1: Snippets of Unassisted page treatment plan viewer

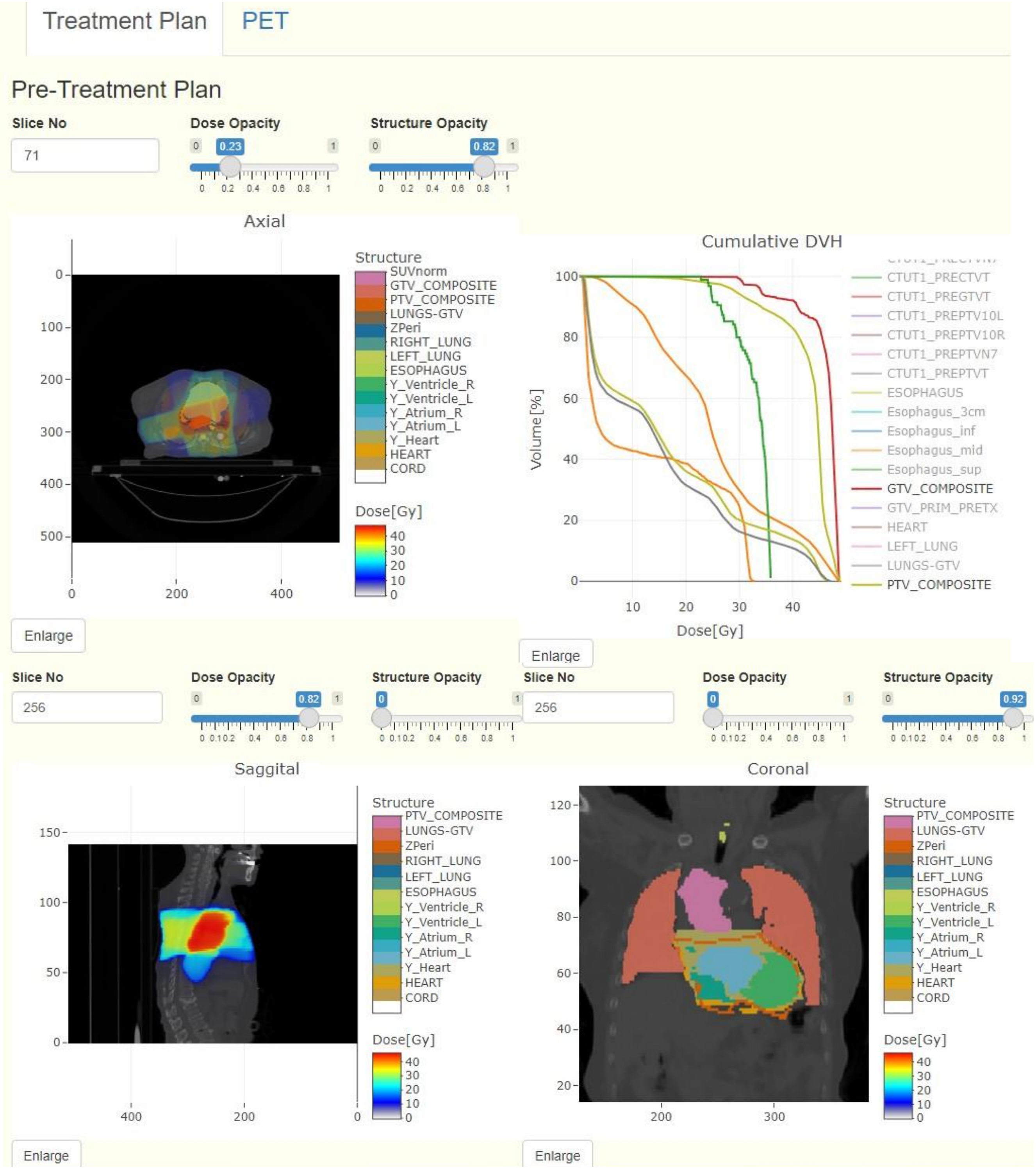

Supplementary Figure 1: Snippets of Unassisted Page treatment plan viewer. The viewer, built on Plotly library, includes Axial, Sagittal, and Coronal view of patients' superimposed CT scan, 3D dose distribution, and Structures, along with their cumulative DVH. Structure and dose values are color coded with discrete and continuous color map, respectively. In addition to the native Plotly controls, the viewer includes three extra controls: slice number input box, dose opacity slider, and structure opacity slider. To demonstrate the controls' functionality, the Axial view has been adjusted to show both dose and structure, the Sagittal to show only dose, and Coronal to show only structure. To demonstrate Plotly's native interactive control, the Coronal View has been zoomed with Plotly's zoom option which has been tweaked to preserve the physical aspect ratio. The native Plotly's option in the cumulative DVH viewer also lets the user select wanted or deselect unwanted histograms. Both NSCLC and HCC Evaluation modules follow the same design.

Supplementary Figure 2. Snippets of Unassisted page PET/MRI viewer

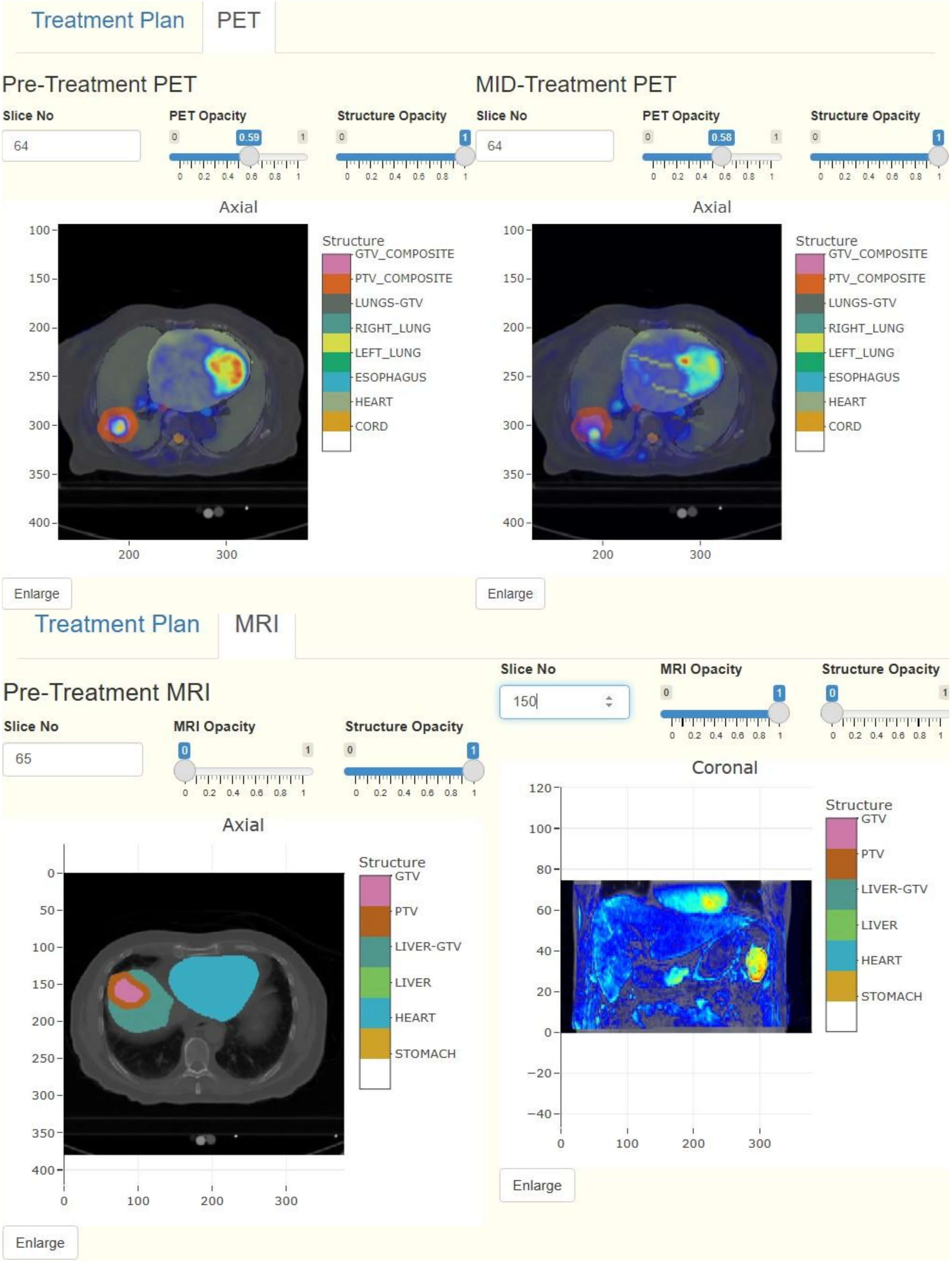

Supplementary Figure 2. Snippets of Unassisted page PET/MRI viewer. The top row shows pre- and mid-treatment PET scans of NSCLC patients in Axial viewer placed in a side-by-side fashion for easy visual comparison. Not shown here are the Sagittal and Coronal Viewers. Bottom row shows pre-treatment MRI scans of HCC patients in Axial and Coronal viewer. Not shown here is the Sagittal Viewer. The viewers superimpose CT scan, PET/MRI image, and structure and include three controls: slice number input box, PET/MRI opacity slider, and structure opacity slider. To demonstrate the controls' functionality, the top row viewers are adjusted to show both PET image and Structure, the Axial MRI viewer is adjusted to show only Structure, and the Coronal MRI viewer to show only MRI scans. The top-row viewers have been zoomed-in using Plotly's native interactive control which has been tweaked to preserve physical aspect ratio.

Supplementary Figure 3. Snippets of AI-assisted Pages showing ARCliDS recommendation, estimated outcome, and feature distribution panels

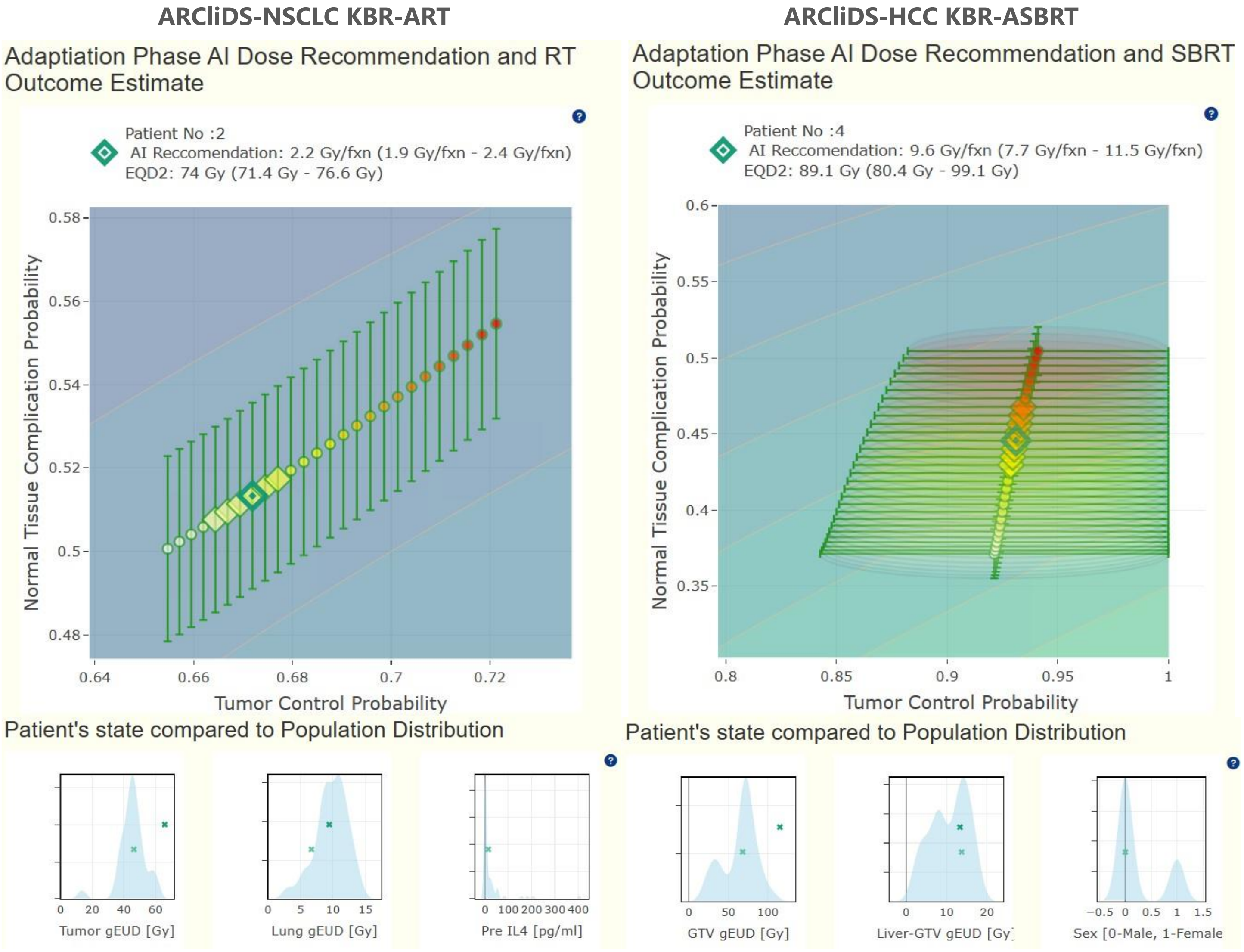

Supplementary Figure 3. Snippets of AI-assisted Pages showing ARCliDS recommendation, estimated outcome, and feature distribution panels. An aggregation of 5 AI-models is included in the Evaluation Module. The AI-recommendation is provided as mean$\pm$sem daily dose fractionation for the adaptive phase of KBR-ART/KBR-ASBRT treatment paradigm. In addition, corresponding total equivalent dose in 2 Gy fractions (EQD2) is provided. Estimated outcome is provided in the outcome space spanned by tumor control probability (TCP) and normal tissue complication probability (NTCP) for adaptive daily dose fractionation value ranging from 1.5 to 4.0 Gy/frac for NSCLC KBR-ART and from 1.0-15.0 Gy/frac for HCC KBR-ASBRT and color-coded continuously from yellow (lowest dose) to red (highest dose). The outcome corresponding to the AI-recommendation are marked with the green diamond markers and the model uncertainty (sem) is given as error bars in both TCP and NTCP direction. Note that data label class-imbalance can give rise to asymmetricity in uncertainty estimate. The background of the outcome space is colored according to the AI reward function, $r = TCP(1 - NTCP)$, which is highest at $(TCP, NTCP) = (1, 0)$. Patient's feature value is presented with population density in the background to provide "whereabouts" about the patient. Not shown here are the feature distribution plots for the remaining 10 features. Help push buttons are included for additional information including feature descriptions. All the viewers are built using Plotly and comes with interactive native controls, such as zooming, selection, etc.

Supplementary Table 2: List of Software and Library

RESOURCE	SOURCE	IDENTIFIER
**Python (v3.9.12)**	Python Software Foundation	RRID:SCR_008394; https://www.python.org/
**NumPy (v1.21.5)**	Python package	RRID:SCR_008633; http://www.numpy.org
**SciPy (v1.7.3)**	Python package	RRID:SCR_008058 http://www.scipy.org/
**Pandas (v1.4.2)**	Python package	RRID:SCR_018214; https://pandas.pydata.org
**PyTorch (v1.11.0)**	Python package	RRID:SCR_018536; https://pytorch.org/
**PyTorch Geometric (v2.0.4)**	Python package	https://pypi.org/project/torch-geometric/; https://github.com/pyg-team/pytorch_geometric
**PyDicom (v2.3.1)**	Python package	RRID:SCR_002573; https://pydicom.github.io/
**Dicompyler-Core (v0.5.5)**	Python package	https://github.com/dicompyler/dicompyler-core
**Scikit-Image (v0.19.3)**	Python package	RRID:SCR_021142; https://scikit-image.org/
**R (v4.2.1)**	R Foundation	RRID:SCR_001905; http://www.r-project.org/
**Shiny (v1.8.0)**	R package	RRID:SCR_001626; http://www.rstudio.com/shiny/
**Reticulate (v1.34.0)**	R package	https://rstudio.github.io/reticulate/
**Plotly (v4.10.3)**	R package	RRID:SCR_013991; https://plotly.com/r/
**Googlesheets4 (v1.1.1)**	R package	https://github.com/tidyverse/googlesheets4
**GoolgeDrive (v2.1.1)**	R package	https://github.com/tidyverse/googledrive
**shinyapps.io (standard subscription)**	Posit	https://www.shinyapps.io/ https://docs.posit.co/shinyapps.io/index.html
**Power Point (v.2401) [office 365]**	Microsoft Crop.	RRID:SCR_023631; https://www.microsoft.com/en-my/microsoft-365/powerpoint

Supplementary Table 3: Evaluators Summary

NSCLC						HCC					
SN	Eval. ID	Exp. (yrs)	Spec.	Inst.	Status	SN	Eval. ID	Exp. (yrs)	Spec.	Inst.	Status
**Residents**											
1	1	4	Breast	1	Complete	13	3	4	General	1	Complete
2	3	4	General	1	Complete	14	6	2	General	2	Complete
3	4	2	General	2	Complete	15	7	3	General	2	Complete
4	6	2	General	2	Complete	16	13	4	General	1	Complete
5	7	3	General	2	Complete	17	D	3	General	2	Didn't Retake
**Resident during training/Physician during Evaluation**											
6	2	5	Prostate	1	Complete	18	12	5	GI	1	Complete
**Physicians**											
7	5	16	CNS	1	Complete	19	5	16	CNS	1	Complete
8	8	21	Prostate	1	Complete	20	10	14	Liver	2	Complete
9	9	17	Lung	1	Complete	21	11	7	GI	1	Complete
10	A	30	GU	1	Didn't retake	22	E	24	GI	2	Didn't retake
11	B	8	Thoracic	1	Didn't Complete	23	F	-	Liver	1	Didn't Take
12	C	-	Lung	1	Didn't Take	24	G	-	GI	1	Didn't Take

Supplementary Table 3: Summary of volunteer evaluators and their relevant information including those who completed the evaluation, didn't take evaluation after initial training/meeting, and those who took the evaluation initially but declined to retake after discovery of a bug and debugging. The names of evaluators and institutions have been de-identified. Four out of 13 evaluators volunteered to take both evaluations hence 17 evaluations by 13 evaluators. Evaluators who completed the evaluation have been de-identified with numbers and rest with alphabets. Note the special classification for the two evaluators who were resident during the initial training/meeting and went on the pass their board certification and formally accepting physician position before completion of evaluation. **Abbreviation: Eval. ID**: Evaluator Identification number, **Exp.**: Experience, **Spec.**: Specialization, **Inst.**: Institution.

Supplementary Figure 4: Decision Adjustment Level

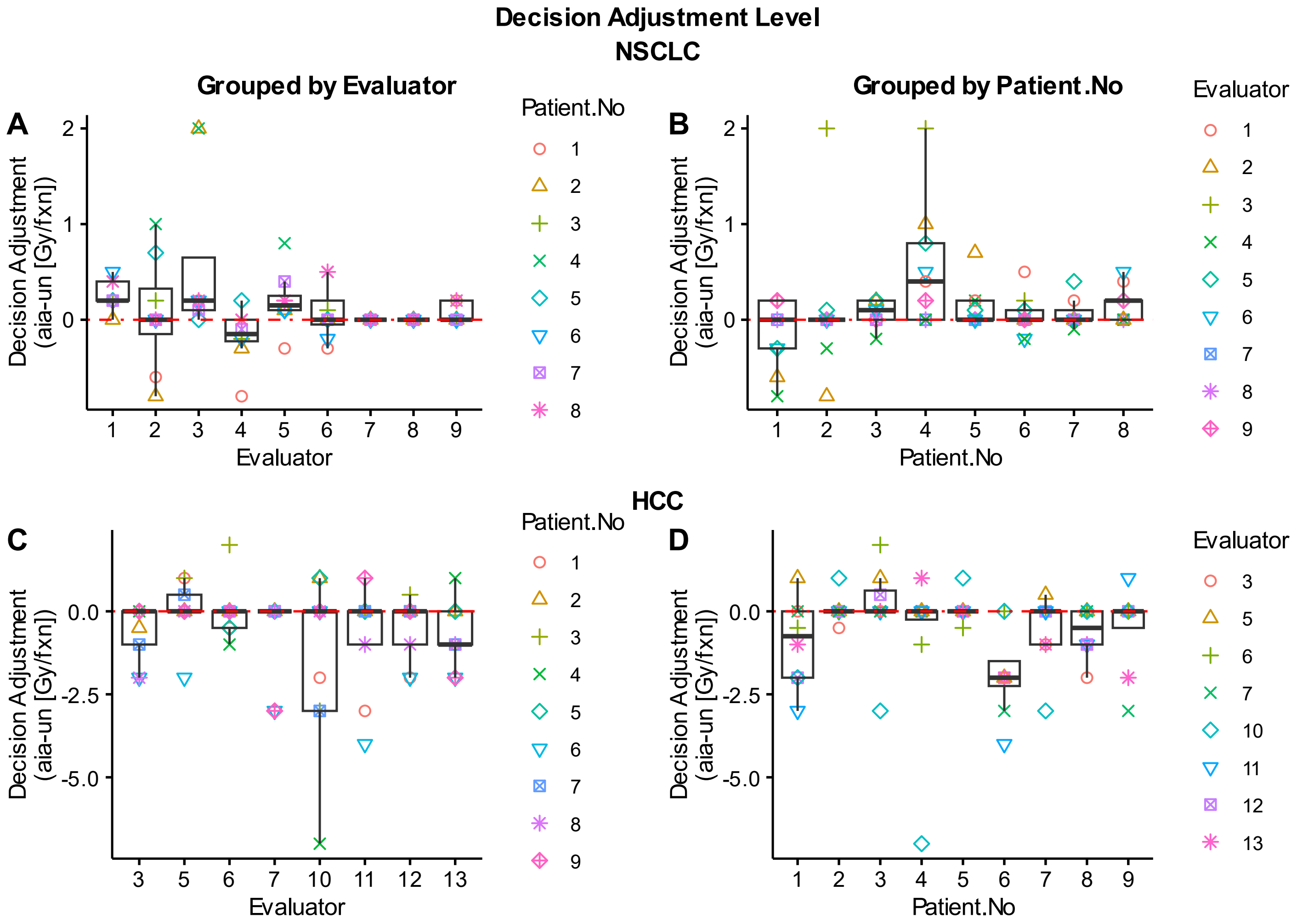

Supplementary Figure 4: Decision Adjustment Level grouped by Evaluators and Patient Number. Box plots a and **b** summarize decision adjustment for NSCLC and **box plots c** and **d** for HCC. The plots grouped by evaluators are marked and color coded by patients and vice versa. Box plots include center line: median, box limits: upper and lower quartiles; whiskers: 1.5x interquartile range; and points: outliers

Supplementary Figure 5: Correlation between Unassisted and AI-assisted Decision and Confidence

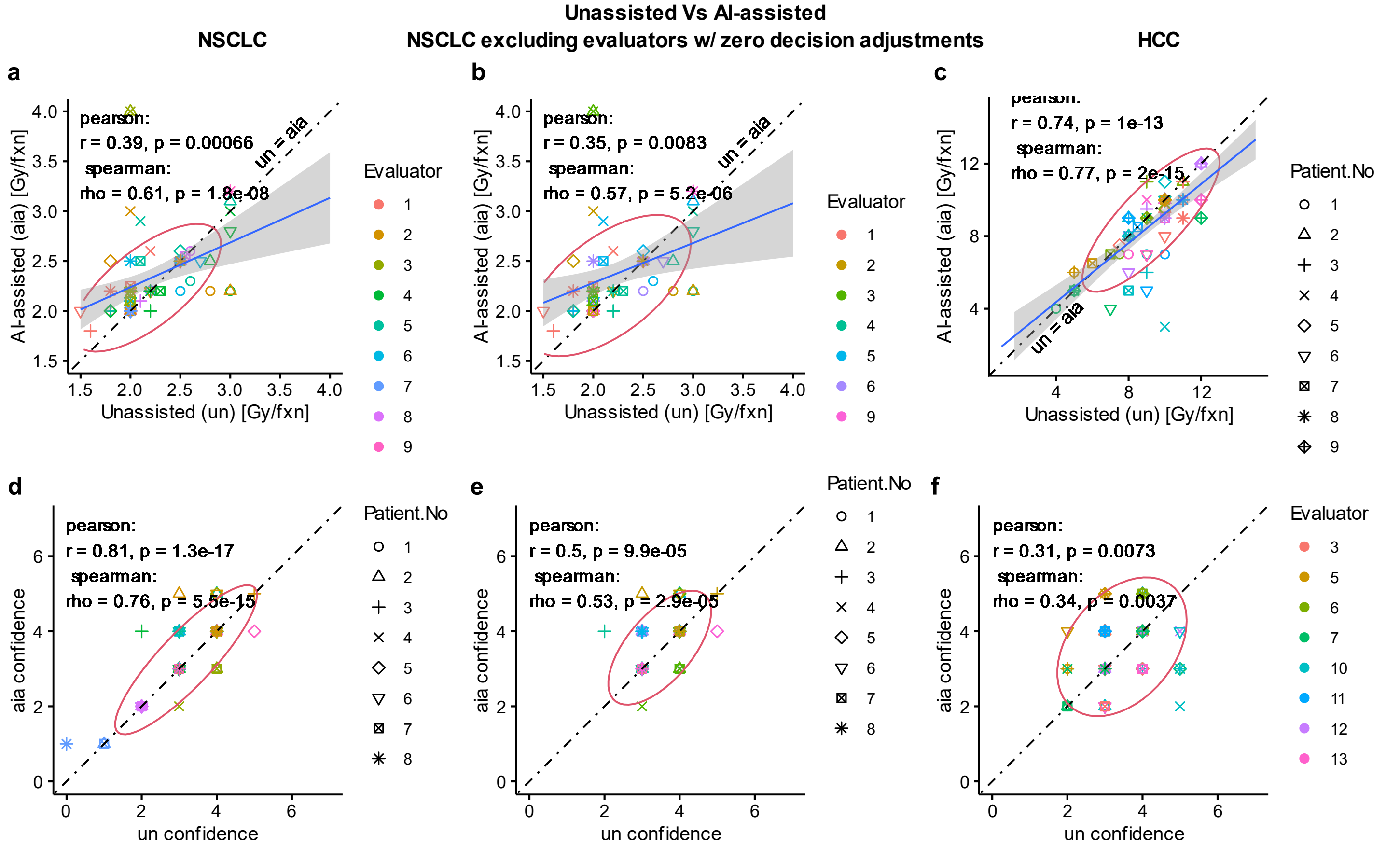

Supplementary Figure 5: Analysis of decision confidence and confidence level. The first, second, and third column of figures corresponds to NSCLC, NSCLC excluding Evaluators with zero decision adjustment, and HCC, respectively, and all the plots are colored by Evaluators and marked coded Patient Number. Note, all evaluators in HCC adjusted at least one of their decisions. **Scatter plots a, b,** and **c** show the relationship between unassisted (un) and AI-assisted decision (aia) and present the Pearson and Spearman correlation coefficient with p-value (two-sided t test), covariance ellipse (95% confidence), a solid linear fit line and uncertainty (95% confidence interval), and the dot-dashed null-hypothesis line $un = aia$ which represents absence of AI-influence. The $ai - un = 0$ line corresponds to complete agreement between unassisted decision and AI-recommendation, whereas $aia - un = 0$ line corresponds to absence of decision change. **2D Scatter plots d**, **e**, and **f** show the relationship between evaluators' unassisted decision (un) confidence and AI-assisted decision (aia) confidence (scaled 0-5, 5 being highest).

We investigated the correlation between un and aia. Under null, un and aia should show perfect (or near perfect) correlation and the tuple (un, aia) should distribute close to the $un = aia$ line; however, as shown in **Figures a and c**, for NSCLC, we found Pearson correlation coefficient (r) of 0.39 ($p < 0.001$) and spearman correlation coefficient (ρ) of 0.61 ($p < 0.001$), and for HCC, $r = 0.74$ ($p < 0.001$) and $\rho = 0.77$ ($p < 0.001$) which are not near perfect. Pearson coefficient represents level of linear relationship between un and aia however, unlike Spearman rank correlation, it is very sensitive to extreme value which is seen from the drastic difference between NSCLC r and ρ. In addition, we investigated evaluators who adjusted at least one of their decisions. As expected, we found a decrease in the correlation value as shown in **Figure b**, i.e. $r = 0.35$ ($p = 0.0083$) and $\rho = 0.57$ ($p < 0.001$). The best fit line for both cases did not align with null hypothesis line $un = aia$. The combination of results from hypothesis testing, frequency analysis, and correlation analysis indicated that AI influences decisions on a case-by-case basis. Then, we investigated the relationship between evaluators self-reported Unassisted decision confidence and AI-assisted decision confidence. Under null (no AI-influence), we would expect $aia\ conf$ and $un\ conf$ to show a perfect (or near perfect) correlation and the tuple $(un\ conf, aia\ conf)$ to distribute close to the $un\ conf = aia\ conf$ line. As shown in **Figures d**, and **f**, we found a positive correlation between $aia\ conf$ and $un\ conf$ decision: for NSCLC, $r = 0.81$ ($p < 0.001$) and $\rho = 0.76$ ($p < 0.001$) and for HCC, $r = 0.31$ ($p = 0.0073$) and $\rho = 0.34$ ($p = 0.0037$). The correlation coefficient is not as strong as we would expect for the null case. Moreover, for NSCLC excluding evaluators with zero decision adjustment, we obtained $r = 0.5$ ($p < 0.001$) and $\rho = 0.53$ ($p < 0.001$), as shown in **Figure e**, which is considerably less than that of the overall NSCLC, consistent with the alternative hypothesis.

Supplementary Figure 6: Individual AI-Trust Level Contribution vs Agreement with AI-recommendation

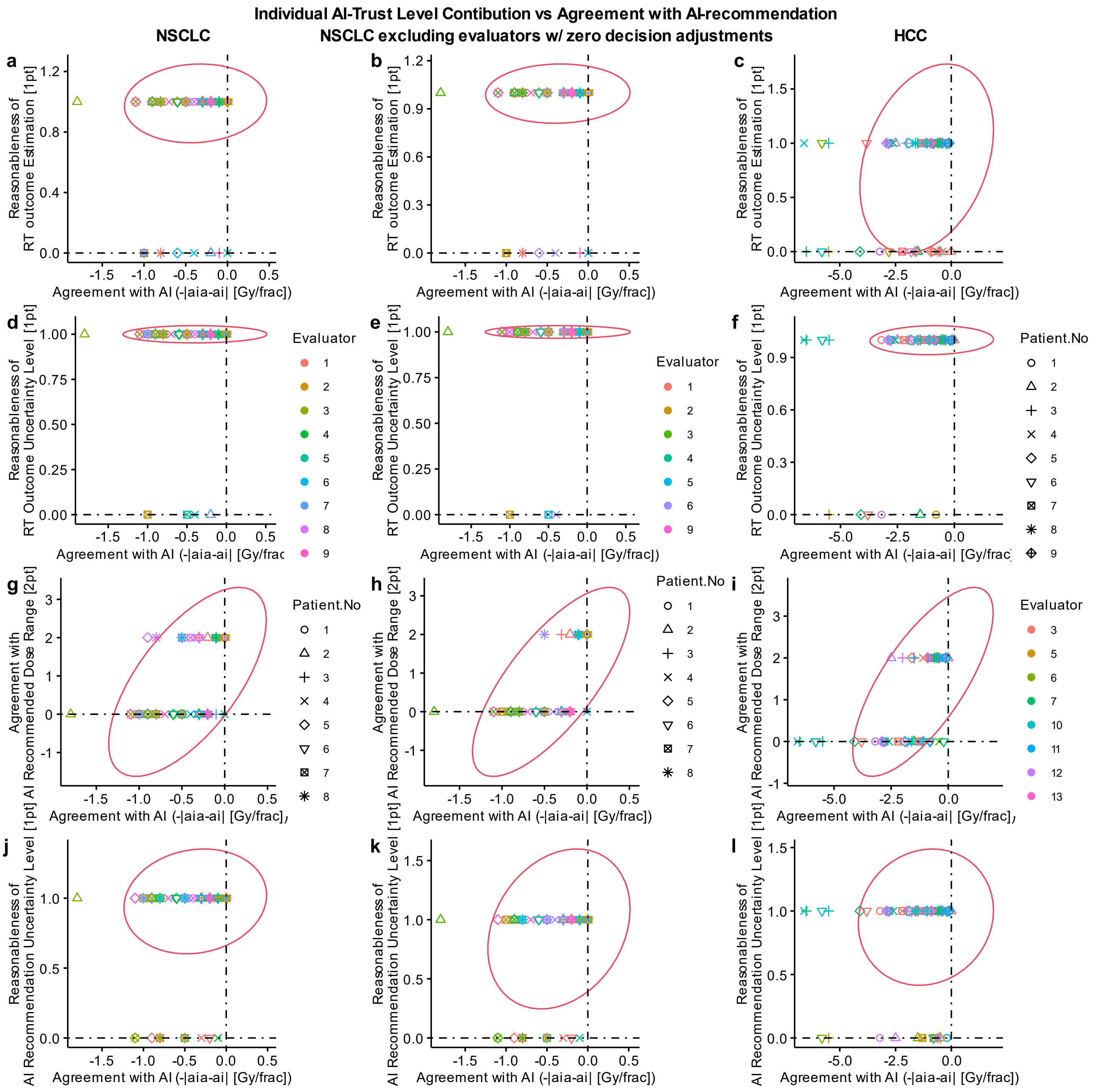

Supplementary Figure 6: Analysis of individual contribution to AI Trust level with respect to agreement between AI-assisted Decision and AI-recommendation. The first, second, and third column of figures corresponds to NSCLC, NSCLC excluding Evaluators with zero decision adjustment, and HCC, respectively, and all the plots are colored by Evaluators and marked coded Patient No. Evaluator's level of trust on AI (scaled 0-5, 5 being the highest) was determined based off of their self-reported agreement with AI's RT outcome estimate (**first two rows**) and AI-recommendation (**last two rows**) which was further divided into average trend (**first and third row**) and uncertainty estimate (**second and last rows**). The individual contributions were Reasonableness of RT outcome Estimation (1 pt), Reasonableness of RT outcome Uncertainty Level (1pt); Agreement with AI recommended Dose range (2pts), and Reasonableness of AI Recommendation Uncertainty Level (1pt). For visual insight, covariance ellipses (95% confidence) are included.

Supplementary Figure 7: Authors summary of Evaluators' Remark for NSCLC.

Pat	Eval	un (Gy/fx)	un conf	Unassisted Remark Summary-NSCLC
1	9	2	4	Concerned about OAR[1] (esophagus)
2	9	2	4	Concerned about OAR[1] (esophagus)
3	2	1.8	5	Concerned about OAR[1] (lung)
	4	2.2	2	Anatomical Adaptation[4] instead of dose adaptation
	9	2	4	Concerned about OAR[1] (esophagus). Disagree with dose escalation.
4	3	2	3	Disagree with KBR-ART Evaluation Phase dose fractionation[2] and potential inconsistency with DVH[3]
	4	3	4	Potential inconsistency with DVH[3], potential dose escalation
	9	2	3	Trouble with PET treatment Planner[5], OAR[1] dose high, potential dose de-escalation
5	4	1.8	3	Positive response by patient during KBR-ART evaluation phase[2]. OAR[1]. Consider other clinical factors.
	7	2.2	3	Positive KBRART Evaluation Phase dose response[2] by patient
6	9	2.5	3	Potential dose escalation if OAR[1] spared
7	9	2	4	Concerned about OAR[1] (esophagus). Potentially dose deescalate
8	7	2	0	Positive KBR-ART evaluation phase dose response[2] by patient. Potential Dose de-escalation.
	9	3	3	OAR[1] seems safe. Potential dose escalation. Not enough knowledge.

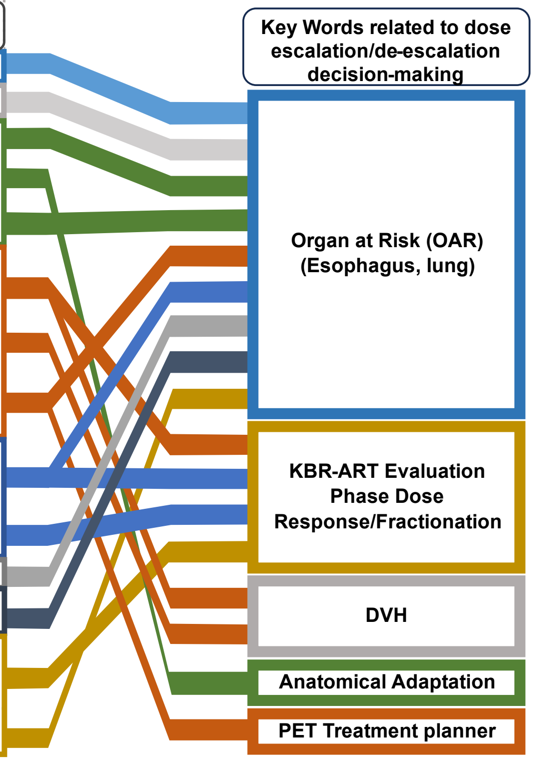

Pat	Eval	un (Gy/fx)	ai (Gy/fx)	aia (Gy/fx)	un conf	aia conf	ai trust	AI-assisted Remark Summary-NSCLC
2	9	2	2.2	2	4	4	3	relation between RTOE and AI-recommendation[2] seems inconsistent, concerned about RTOE-NTCP[1] accuracy
3	9	2	2.1	2	4	4	4	concerned about RTOE-NTCP[1] accuracy w.r.t OAR[3](esophagus)
4	3	2	2.9	4	3	2	2	relation between RTOE and AI-recommendation[2] seems inconsistent; AI-recommendation must have been higher according to the RTOE
	4	3		3	4	4	4	concerned about RTOE-NTCP[1] accuracy: low rise (NTCP) over run (dose)
	6	2		2.5	3	3	1	concerned about RTOE-NTCP[1] accuracy: low rise (NTCP) over run (dose)
5	4	1.8	3.1	2	3	3	3	uncertain about RTOE-NTCP[1] modeling w.r.t to OAR[3] lung, esophagus, bronchus, and incorporation of chemo information[7]
	9	2		2	5	4	3	concerned about OAR[3] (esophagus) toxicity for the AI recommendation
6	4	3	2.2	2.8	3	3	3	concerned about RTOE[4] error bars
	9	2.5		2.5	3	3	3	relation between RTOE and AI-recommendation[2] seems inconsistent
7	2	2	3	2	4	4	1	target volumes[5] are large for hypofractionation
	3	2		2.1	4	3	3	relation between RTOE and AI-recommendation[2] seems inconsistent
8	3	2	3	2.2	4	4	2	decision adjustment made based on RTOE[4], bypassing AI-recommendation
	7	2		2	0	1	2	Confirmation bias[6] on prior clinical trials showing no benefits of dose escalation
	9	3		3.2	3	3	3	OAR[3] (esophagus) seems safe. Potential dose escalation.

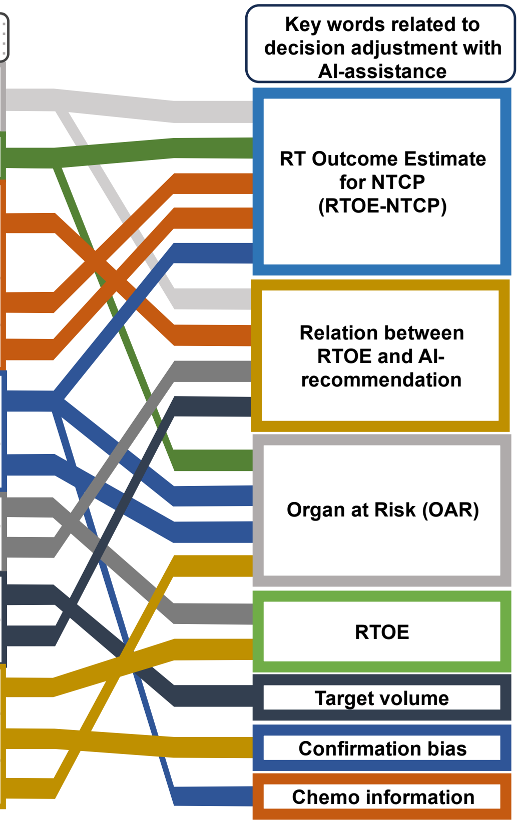

Supplementary Figure 7: Authors summary of Evaluators' Remark for NSCLC. The tables on the left summarize Evaluators' Remark and present corresponding decision, decision confidence, and AI trust level for Un-assisted and AI-assisted Phase. To present a statistic the repeated keywords are identified and displayed in boxes in the right columns. Following Shankey Diagram's approach, we matched the evaluator's remark to the keyword boxes. The box height is proportional to keyword frequency, and they are sorted from the most frequent to least frequent. Evaluators' full remarks are included in the supplementary material. **Abbreviation- Pat:** Patient No**; Eval:** Evaluator**; un:** Unassisted Decision**; ai:** AI recommendation**; aia**: AI-assisted Decision; **un conf**: Unassisted Decision Confidence; **aia conf**: AI-assisted Decision Confidence; **ai trust**: AI recommendation trust level

Supplementary Figure 8: Authors summary of Evaluators' Remark for HCC.

Pat	Eval	un (Gy/fx)	un conf	Unassisted Remark Summary-HCC
1	11	10	4	Negative KBR-ART Evaluation Phase response[3] (LF[2]), aim for 100 BED[1], CP A disease[4]
	13	8	3	potential dose de-escalation
4	11	9	4	Positive KBR-ART Evaluation Phase response[3] (LF[2]), aim for 100 BED[1]
5	11	9	3	concern with OAR[5] (heart), dose deescalate, aim for 100 BED[1]
6	11	9	3	aim for 100 BED[1]
7	11	8.5	3	Large tumor[6], positive Evaluation Phase response[3] (LF[2]), aim for >72 BED[1], CP B disease[4]
8	11	11	3	Positive KBR-ART Evaluation Phase response[3]
9	10	8	4	Bad Liver Function[2]
	11	8	3	CP B Disease[4], aim for BED >100[1]

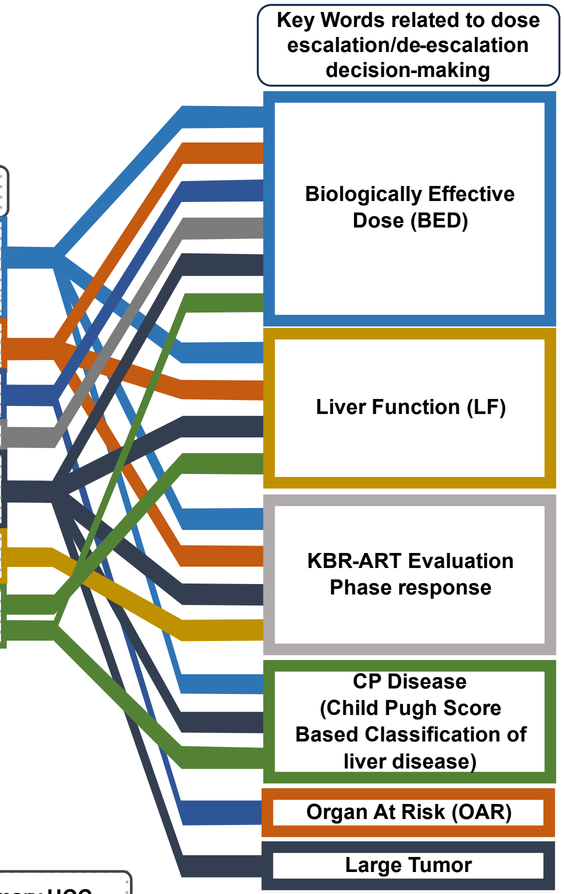

Pat	Eval	un (Gy/fx)	ai (Gy/fx)	aia (Gy/fx)	un conf	aia conf	ai trust	AI-assisted Remark Summary-HCC
1	11	10	6.8	7	4	4	4	Agreement with AI[2], lower dose to BED[4]>72
2	10	8	9.5	9	3	2	4	Decision Adjustment with RTOE[1]
3	11	10	11.5	10	3	4	5	Disagreement with AI[2], concerned and uncertain if OAR[3] safe
4	10	10	9.6	3	5	2	3	relation between RTOE and AI-recommendation seems inconsistent;
	11	9		9	4	5	5	Agreement with AI[2]
5	10	10	9.1	11	4	3	3	decision adjustment made based on RTOE[1] bypassing AI-recommendation
6	7	7	4.2	4	3	2	3	decision adjustment made based on RTOE[1] while agreeing with AI-recommendation, Steep Rise (NTCP) over run (dose) seems low
	10	10		10	5	4	2	decision adjustment made based on RTOE[1] bypassing AI-recommendation; Steep Rise (NTCP) over run (dose) seems low
	11	9		5	3	3	2	decision adjustment made based on RTOE[1] while agreeing with AI-recommendation
7	10	8	7.8	5	4	3	3	decision adjustment made based on RTOE[1] bypassing AI-recommendation; Steep Rise (NTCP) over run (dose) seems low
9	11	8	9.1	9	3	4	5	Agreement with AI[2]

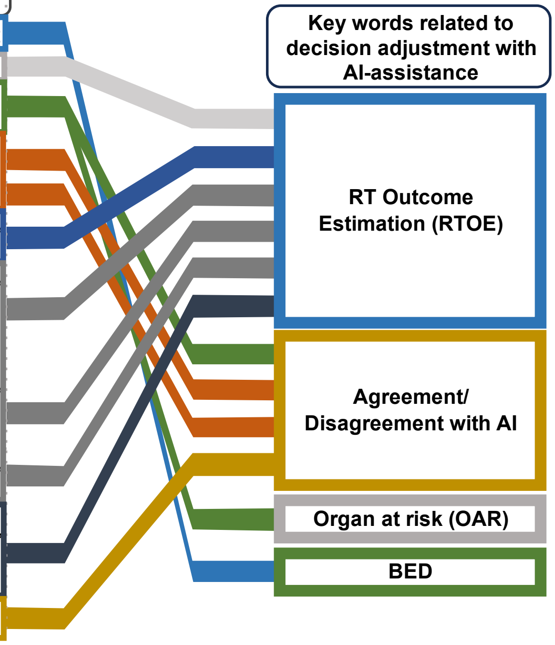

Supplementary Figure 8: Authors summary of Evaluators' Remark for HCC. The tables on the left summarize Evaluators' Remark and present corresponding decision, decision confidence, and AI trust level for Un-assisted and AI-assisted Phase. To present a statistic the repeated keywords are identified and displayed in boxes in the right columns. Following Shankey Diagram's approach, we matched the evaluator's remark to the keyword boxes. The box height is proportional to keyword frequency, and they are sorted from the most frequent to least frequent. Evaluators' full remarks are included in the supplementary material. **Abbreviation- Pat:** Patient No**; Eval:** Evaluator**; un:** Unassisted Decision**; ai:** AI recommendation**; aia**: AI-assisted Decision; **un conf**: Unassisted Decision Confidence; **aia conf**: AI-assisted Decision Confidence; **ai trust**: AI recommendation trust level
