# Supplementary material for: Intricacies of human–AI interaction in dynamic decision-making for precision oncology
Source: Nat Commun. 2025 Jan 29;16:1138. doi: 10.1038/s41467-024-55259-x (PMC11779952; doi:10.1038/s41467-024-55259-x)
Supplement: Supplementary file 3 — Reporting Summary [file 41467_2024_55259_MOESM3_ESM.pdf]

nature portfolio

Corresponding author(s): Dipesh Niraula

Last updated by author(s): 11/27/2024

Reporting Summary

Nature Portfolio wishes to improve the reproducibility of the work that we publish. This form provides structure for consistency and transparency in reporting. For further information on Nature Portfolio policies, see our Editorial Policies and the Editorial Policy Checklist.

Statistics

For all statistical analyses, confirm that the following items are present in the figure legend, table legend, main text, or Methods section.

n/a	Confirmed	
☐	☒	The exact sample size (*n*) for each experimental group/condition, given as a discrete number and unit of measurement
☐	☒	A statement on whether measurements were taken from distinct samples or whether the same sample was measured repeatedly
☐	☒	The statistical test(s) used AND whether they are one- or two-sided<br>*Only common tests should be described solely by name; describe more complex techniques in the Methods section.*
☐	☒	A description of all covariates tested
☐	☒	A description of any assumptions or corrections, such as tests of normality and adjustment for multiple comparisons
☐	☒	A full description of the statistical parameters including central tendency (e.g. means) or other basic estimates (e.g. regression coefficient) AND variation (e.g. standard deviation) or associated estimates of uncertainty (e.g. confidence intervals)
☐	☒	For null hypothesis testing, the test statistic (e.g. *F*, *t*, *r*) with confidence intervals, effect sizes, degrees of freedom and *P* value noted<br>*Give P values as exact values whenever suitable.*
☒	☐	For Bayesian analysis, information on the choice of priors and Markov chain Monte Carlo settings
☒	☐	For hierarchical and complex designs, identification of the appropriate level for tests and full reporting of outcomes
☒	☐	Estimates of effect sizes (e.g. Cohen's *d*, Pearson's *r*), indicating how they were calculated

Our web collection on statistics for biologists contains articles on many of the points above.

Software and code

Policy information about availability of computer code

Data collection	The prospective MD evaluation data was collected using web application written in R (v4.2.1) using shiny(v1.8.0) and hosted in shinyapps.io servers. The backend of the web app was developed in Python (v3.9.12) using packages NumPy (v1.21.5), SciPy (v1.7.3), Pandas (v1.4.2), PyTorch (v1.11.0), PyTorch Geometric (v2.0.4), PyDicom (v2.3.1), Dicompyler-Core (v0.5.5), Scikit-Image (v0.19.3), Reticulate (v1.34.0), and Plotly (v4.10.3). The data were automatically saved in online googlesheets which were facilitated by R packages: Goolgesheets4 (v1.1.1) and GoogleDrive (v2.1.1).
Data analysis	Statistical Analysis was performed in R (v4.2.1). ICC were calculated with irr (v 0.84.1) and plots were generated with ggpubr (v0.6.0).

For manuscripts utilizing custom algorithms or software that are central to the research but not yet described in published literature, software must be made available to editors and reviewers. We strongly encourage code deposition in a community repository (e.g. GitHub). See the Nature Portfolio guidelines for submitting code & software for further information.

Data

Policy information about availability of data

All manuscripts must include a data availability statement. This statement should provide the following information, where applicable:

- Accession codes, unique identifiers, or web links for publicly available datasets
- A description of any restrictions on data availability
- For clinical datasets or third party data, please ensure that the statement adheres to our policy

The source data (evaluator's decision data) are provided with this paper. IRB restrictions apply to the patient's clinical data and are not publicly available. For this study, the data were obtained from the University of Michigan under data sharing protocol. To obtain the data, a formal data sharing application must be submitted to the Department of Radiation Oncology, University of Michigan.

Research involving human participants, their data, or biological material

Policy information about studies with human participants or human data. See also policy information about sex, gender (identity/presentation), and sexual orientation and race, ethnicity and racism.

Reporting on sex and gender	The clinical training/testing data used in the AI models are retrospectively collected. Information regarding sex are provided as part of the patient demographics. MD Evaluators' sex/gender information were not considered in the study design; it is our understanding based on the current literature and the methodology used to analyze decision making that no dependency or associated bias is known.
Reporting on race, ethnicity, or other socially relevant groupings	This study did not collect information or investigate aspect related to race/ethnicity or other social factors.
Population characteristics	The AI training/testing population characteristics and MD evaluators demographics are provided in the supplementary.
Recruitment	No patient recruitment. MD evaluators were recruited via a combination of group emails and in-person invitations to the members of the radiation oncology department at Moffitt Cancer center and Michigan Medicine. The evaluators volunteered for this study, as such, there is a possibility of self-selection bias. We made effort in recruiting a diverse group of evaluators; the evaluators consisted of both physicians and residents from two institutions located in two states with a variety of specializations and a range of experience. However, there is still a possibility for selection/sampling bias influencing the result of this study.
Ethics oversight	The study is approved under IRB Moffitt Cancer Center- MCC# 20750

Note that full information on the approval of the study protocol must also be provided in the manuscript.

Field-specific reporting

Please select the one below that is the best fit for your research. If you are not sure, read the appropriate sections before making your selection.

☐ Life sciences ☒ Behavioural & social sciences ☐ Ecological, evolutionary & environmental sciences

For a reference copy of the document with all sections, see nature.com/documents/nr-reporting-summary-flat.pdf

Behavioural & social sciences study design

All studies must disclose on these points even when the disclosure is negative.

Study description	Qualitative experimental research, mainly quantitative data and some qualitative data (evaluators' text remark)
Research sample	Evaluators were medical experts (physicians and residents) in radiation oncology from investigator's affiliated institutes: Moffitt Cancer Center and Michigan Medicine. A description of evaluators' experience and specialty are provided in supplementary table S2. We enrolled 13 evaluators out of which, 4 evaluators volunteered in both NSCLC and HCC studies, resulting in a total of 17 completed evaluations (9 NSCLC and 8 HCC). To limit the evaluation time to under an hour, we selected 8 treated patients for NSCLC and 9 for HCC, resulting in a total of 144 sets of evaluations (72 from NSCLC and 72 from HCC). Evaluation for each patient consisted of 8 required inputs and 2 optional remarks, resulting in up to a total of 1440 data points.
Sampling strategy	Data collection for the interaction experiments was performed prospectively. No sample size calculation was performed. We advertised the study via a combination of group emails and in-person invitation. The sample size in this study was determined by the number of willing experts from the two institutes.
Data collection	Evaluators took the web-based evaluation remotely on their personal device via a web browser. The researchers were not present during evaluation. A user account system was added so the evaluation could be completed in multiple sessions if needed, and automatic saving were enabled for all user inputs. To homogenize evaluators' first impression of AI we conducted a pre-evaluation

information session with the evaluators, in which, we played to them training videos and demonstrated sample evaluation followed by a question-and-answer round. The evaluation modules randomly initialized ordering of patients, so that each evaluator would interact with the same group of patients in a different ordering. We chose a sequential design, where a unit of evaluation consisted of unassisted phase followed by AI-assisted phase. Evaluation modules isolated the decision-making process by restricting evaluators to revisit the Unassisted Phase once they have seen the AI-recommendation and by excluding Unassisted Phase decisions from the AI-assisted Phase page. Researchers provided technical support when requested.

Timing

The data collection period lasted for 5 months which started on march 2023 and ended on July 2023.

Data exclusions

No data were excluded from this study.

Non-participation

Initially, a total of 20 volunteers showed interest and initial pre-evaluation information sessions were conducted, mostly in a one-on-one virtual meeting, two in-person meetings, and one group virtual meeting with three volunteers. Initially, we found a bug in the software, which was corrected, and the five evaluators who had taken the evaluation were requested to retake the evaluation to which only two out of five agreed, and thus the remaining 3 evaluations were discarded. Similarly, three evaluators didn't follow up and one evaluator only completed evaluation for one patient. In total, 13 evaluators completed the evaluation, and 4 evaluators volunteered to take both NSCLC and HCC evaluation, with a total of 17 completed evaluations (9 NSCLC, and 8 HCC). The details are included in supplementary table S2.

Randomization

The order of presentation of the treatment plans was randomized but the participants were assigned into NSCLC and HCC group based on their area of expertise or by their preference.

Reporting for specific materials, systems and methods

We require information from authors about some types of materials, experimental systems and methods used in many studies. Here, indicate whether each material, system or method listed is relevant to your study. If you are not sure if a list item applies to your research, read the appropriate section before selecting a response.

Materials & experimental systems

n/a	Involved in the study
☒	☐ Antibodies
☒	☐ Eukaryotic cell lines
☒	☐ Palaeontology and archaeology
☒	☐ Animals and other organisms
☐	☒ Clinical data
☒	☐ Dual use research of concern
☒	☐ Plants

Methods

n/a	Involved in the study
☒	☐ ChIP-seq
☒	☐ Flow cytometry
☒	☐ MRI-based neuroimaging

Clinical data

Policy information about clinical studies

All manuscripts should comply with the ICMJE guidelines for publication of clinical research and a completed CONSORT checklist must be included with all submissions.

Clinical trial registration

NSCLC: NCT01190527, HCC: NCT01519219, NCT01522937, and NCT0246083514

Study protocol

Moffitt Cancer Center- MCC# 20750

Data collection

The AI model training is retrospective but the evaluation is prospective.

Outcomes

NSCLC: Local control and Radiation induced Pneumonitis. HCC: Local control and Liver Function.

Plants

Seed stocks

Report on the source of all seed stocks or other plant material used. If applicable, state the seed stock centre and catalogue number. If plant specimens were collected from the field, describe the collection location, date and sampling procedures.

Novel plant genotypes

Describe the methods by which all novel plant genotypes were produced. This includes those generated by transgenic approaches, gene editing, chemical/radiation-based mutagenesis and hybridization. For transgenic lines, describe the transformation method, the number of independent lines analyzed and the generation upon which experiments were performed. For gene-edited lines, describe the editor used, the endogenous sequence targeted for editing, the targeting guide RNA sequence (if applicable) and how the editor was applied.

Authentication

Describe any authentication procedures for each seed stock used or novel genotype generated. Describe any experiments used to assess the effect of a mutation and, where applicable, how potential secondary effects (e.g. second site T-DNA insertions, mosiacism, off-target gene editing) were examined.
